# Role of systematic lymphadenectomy as part of primary debulking surgery for optimally cytoreduced advanced ovarian cancer: Reappraisal in the era of radical surgery

**DOI:** 10.18632/oncotarget.13696

**Published:** 2016-11-29

**Authors:** Kyung Jin Eoh, Jung-Yun Lee, Jung Won Yoon, Eun Ji Nam, Sunghoon Kim, Sang-Wun Kim, Young Tae Kim

**Affiliations:** ^1^ Department of Obstetrics and Gynecology, Institute of Women's Life Medical Science, Yonsei University College of Medicine, Seoul, Korea

**Keywords:** lymphadenectomy, advanced epithelial ovarian cancer, primary debulking surgery, optimal cytoreductive surgery, lymph node dissection

## Abstract

The prognostic significance of pelvic and para-aortic lymphadenectomy during primary debulking surgery for advanced-stage ovarian cancer remains unclear. This study aimed to evaluate the survival impact of lymph node dissection (LND) in patients treated with optimal cytoreduction for advanced ovarian cancer. Data from 158 consecutive patients with stage IIIC–IV disease who underwent optimal cytoreduction (<1 cm) were obtained via retrospective chart review. Patients were classified into two groups: (1) lymph node sampling (LNS), node count <20; and (2) LND, node count ≥20. Progression-free (PFS) and overall survival (OS) were analyzed using the Kaplan–Meier method. Among the included patients, 96 and 62 patients underwent LND and LNS as primary debulking surgery, respectively. There were no differences in the extent of debulking surgical procedures, including extensive upper abdominal surgery, between the groups. Patients who underwent LND had a marginally significantly improved PFS (P = 0.059) and significantly improved OS (P < 0.001) compared with those who underwent LNS. In a subgroup with negative lymphadenopathy on preoperative computed tomography scans, revealed LND correlated with a better PFS and OS (P = 0.042, 0.001, respectively). Follow-ups of subsequent recurrences observed a significantly lower nodal recurrence rate among patients who underwent LND. A multivariate analysis identified LND as an independent prognostic factor for PFS (hazard ratio [HR], 0.629; 95% confidence interval [CI], 0.400–0.989) and OS (HR, 0.250; 95% CI, 0.137–0.456). In conclusion, systematic LND might have therapeutic value and improve prognosis for patients with optimally cytoreduced advanced ovarian cancer.

## INTRODUCTION

More than two-thirds of patients with epithelial ovarian cancer (EOC) have advanced disease at the time of diagnosis; hence, EOC remains a major cause of gynecologic cancer-related mortality [1]. Currently, the primary standard treatment for advanced-stage EOC comprises debulking surgery and adjuvant taxane- and platinum-based chemotherapies [2]. Radical debulking surgery is a critical treatment strategy for advanced ovarian cancer, and several studies have supported the importance of maximal cytoreductive surgical efforts to minimize residual disease [3, 4].

Notably, the role of systematic lymph node dissection (LND) in the treatment of stage IIIC–IV ovarian cancer remains controversial because this procedure does not influence the surgical stage and its therapeutic benefit is uncertain [5–7]. Consensus has not yet been reached regarding the efficacy of this procedure, despite the status of retroperitoneal lymph node metastasis was reported as a major risk factor for poor prognosis [8–10]. Current National Comprehensive Cancer Network (NCCN) guidelines do not recommend systematic LND other than the removal of suspicious and/or enlarged nodes in patients with advanced disease. In addition, 2 previous randomized controlled trials (RCTs) failed to identify a significant benefit of systematic LND for overall survival (OS) [5, 11], whereas retrospective studies have demonstrated the potential favorable impact of this procedure on OS [9, 10, 12, 13]].

Previous studies were, however, performed before radical surgery was generally accepted as a standard therapy for advanced ovarian cancer [14, 15]. Therefore, the role of systematic LND merits further investigation in the era of radical surgery. The present study aimed to evaluate the survival impact of systematic LND as part of optimal primary debulking surgery for the treatment of advanced ovarian cancer.

## RESULTS

Among 274 patients who were diagnosed with advanced epithelial ovarian cancer at our institution from 2006 to 2015, optimal cytoreduction was achieved in 175 (63.9%); of these, the lymphadenectomy status could be identified in 158 patients. The clinicopathological characteristics of the patients included in this study are listed in Table 1. A total of 158 consecutive patients with optimally cytoreduced primary International Federation of Gynecology and Obstetrics (FIGO) stage IIIC and IV disease who had received adjuvant platinum-based chemotherapy during the study period were analyzed. Of these patients, 62 (39.2%) underwent lymph node sampling (LNS) and 96 (60.8%) underwent LND (including both pelvic and para-aortic lymphadenectomy) as part of primary debulking surgery. The groups were similar with respect to age, preoperative cancer antigen (CA) 125 level, suspected lymph node metastasis on preoperative computed tomography (CT) scan, histology, FIGO stage, and grade. In addition, the groups did not differ with regard to the radicality of surgical procedures, including bowel resection, diaphragm resection, peritonectomy, and video-assisted thoracoscopic surgery (VATS), but did differ significantly in terms of the number of dissected lymph nodes (P < 0.001; Table 2). Furthermore, the rates of no gross residual disease after debulking surgery did not differ significantly between the groups (P = 0.141).

**Table 1:** Characteristics of patients in the study

Variables	LNS (n = 62)	LND (n = 96)	P-value
Age, median (range)	53 (27-81)	55 (32-78)	0.359
BMI (kg/m^2^)	23.1 (11.6-35.3)	22.5 (17.5-31.4)	0.192
Preoperative serum CA 125 (U/mL)	1643.6 (16.3–17223.1)	1637.1 (13.5–11582.7)	0.987
Suspected LN metastasis on CT scan			
Yes	30 (48.4%)	56 (58.3%)	0.22
No	32 (51.6%)	40 (41.7%)	
Histology			
Serous	45 (72.6%)	85 (88.5%)	0.182
Mucinous	0	1 (1.0%)	
Endometrioid	7 (11.3%)	4 (4.2%)	
Clear cell	3 (4.8%)	3 (3.1%)	
Mixed	3 (4.8%)	2 (2.1%)	
Undifferentiated	1 (1.6%)	0	
Unknown	3 (4.8%)	1 (1.0%)	
Stage			
IIIC	47 (75.8%)	74 (77.1%)	0.853
IV	15 (24.2%)	22 (22.9%)	
Tumor grade			
Grade 1	1 (1.6%)	6 (6.3%)	0.209
Grade 2	21(33.9%)	39 (40.6%)	
Grade 3	40 (64.5%)	51 (53.1%)	
LN metastasis			
Yes	33 (53.2%)	70 (72.9%)	0.011
No	29 (46.8%)	26 (27.1%)	
Residual disease			
NGR	11 (17.7%)	28 (29.2%)	0.141
R < 0.5 cm	30 (48.4%)	33 (34.4%)	
R < 1 cm	21 (33.9%)	35 (36.5%)	

LNS, lymph node sampling; LND, lymph node dissection; SD, standard deviation; N/A, not available; LN, lymph node, NGR, no gross residual disease; R, residual disease

**Table 2:** Characteristics of surgical procedures applied to patients in this study

	LNS (n = 62)	LND (n = 96)	P
Number of resected LNs	10 (0–19)	37 (20–97)	<0.001
Radical surgery			
Bowel resection	15 (24.2%)	19 (19.8%)	0.511
Diaphragm resection	14 (22.6%)	22 (22.9%)	1
Peritonectomy	24 (38.7%)	32 (33.3%)	0.49
VATS	4 (6.5%)	11 (11.5%)	0.294
Ureter resection	3 (4.8%)	2 (2.1%)	0.381
Liver resection	4 (6.5%)	3 (3.1%)	0.434
Splenectomy	2 (3.2%)	8 (8.3%)	0.318
Cholecystectomy	4 (6.5%)	2 (2.1%)	0.212
Residual disease			
NGR	11 (17.7%)	28 (29.2%)	0.141
R < 0.5 cm	30 (48.4%)	33 (34.4%)	
R < 1 cm	21 (33.9%)	35 (36.5%)	

LNS, lymph node sampling; LND, lymph node dissection; LN, lymph node; VATS, video-assisted thoracoscopic surgery; NGR, no gross residual disease; R, residual disease

**Figure 1:**
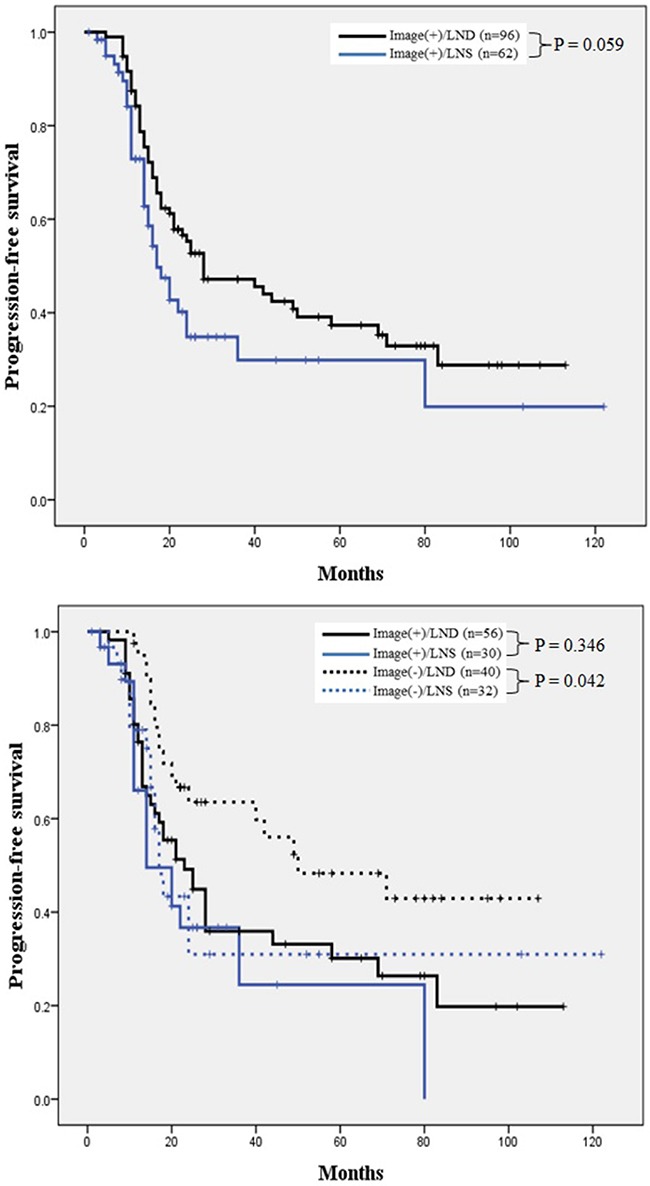
Comparison of PFS in patients who underwent LNS and LND **A**. Overall analysis of PFS. Patients who underwent LND had a favorable PFS with marginal significance (P = 0.059). **B**. Subgroup analysis of PFS according to lymphadenopathy status on preoperative CT scan. In the Image(-) subgroup, LND group showed significantly longer PFS than the LNS groups [log-rank test: Image(+)/LND vs. Image(+)/LNS, P = 0.346; Image(-)/LND vs. Image(-)/LNS, P = 0.042]. PFS, progression-free survival; LNS, lymph node sampling; LND, lymph node dissection.

**Figure 2:**
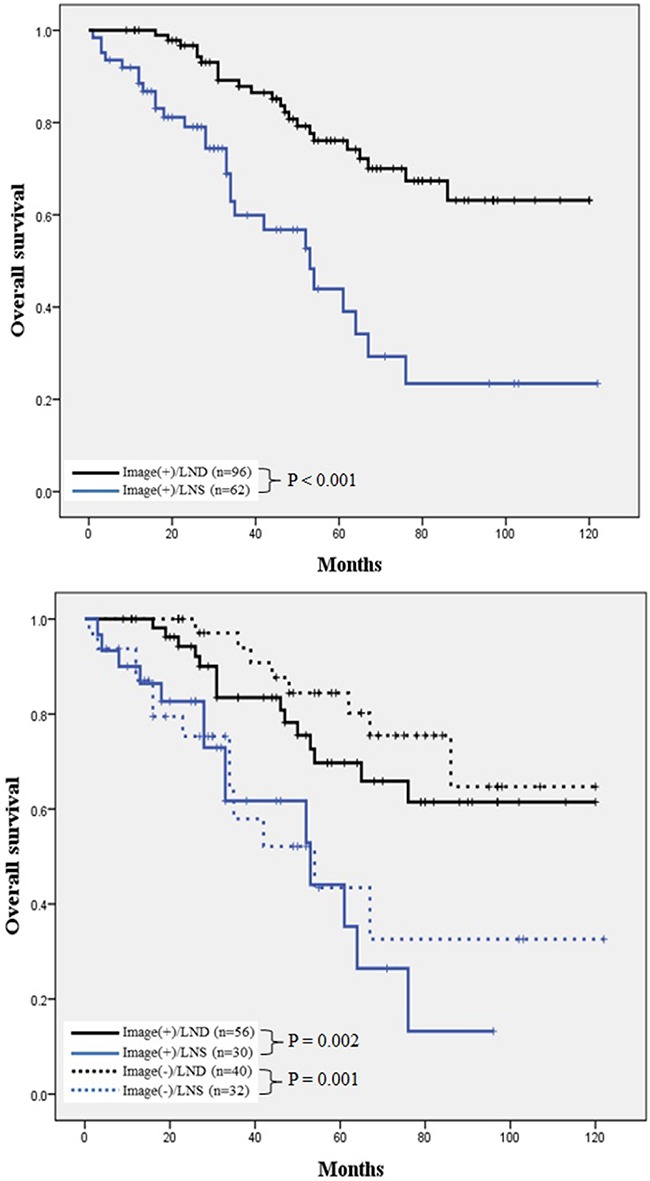
Comparison of OS in patients who underwent LNS and LND **A**. Overall analysis of OS. Patients who underwent LND had a significantly favorable OS, compared to those treated with LNS (P < 0.001). **B**. Subgroup analysis of OS according to lymphadenopathy status on preoperative CT scan. Both LND groups had a significantly longer OS relative to the LNS groups, regardless of the preoperative suspected lymphadenopathy status [log-rank test: Image(+)/LND vs. Image(+)/LNS, P = 0.002; Image(-)/LND vs. Image(-)/LNS, P = 0.001]. OS, overall survival; LNS, lymph node sampling; LND, lymph node dissection.

A Kaplan–Meier survival analysis indicated an apparently favorable progression-free survival (PFS) in the LND group, compared to the LNS group, and this difference was marginally significant (P = 0.059). In the subgroup analysis, LND correlated significantly with a better PFS when compared with LNS among patients with negative lymphadenopathy on a preoperative CT scan (P = 0.042; Figure 1). In addition, patients who underwent LND had a significantly improved OS when compared to those who underwent LNS (P < 0.001), and a subgroup analysis found that LND correlated with a significantly longer OS, regardless of the suspected lymphadenopathy status on a preoperative CT scan (P = 0.001; Figure 2).

A comparison of the characteristics of patients who experienced subsequent recurrence is shown in Table 3. Although the recurrence rates did not differ significantly between the LNS and LND groups (54.8% vs. 58.3%, P = 0.665), a comparison of the recurrence sites revealed a significantly higher nodal recurrence rate in patients who underwent LNS, compared to those who underwent LND (61.8% vs. 33.9%, P = 0.01). In a subgroup analysis, LND correlated with a lower nodal recurrence rate, regardless of the suspected lymphadenopathy status determined by preoperative CT scan (LNS vs. LND: 61.1% vs. 32.4% in the Image(+) subgroup, P = 0.043; 62.5% vs. 36.8% in the Image(-) subgroup, P = 0.13).

**Table 3:** Characteristics of recurrence

	LNS (n = 62)	LND (n = 96)	P	Image(+)/LNS(n = 30)	Image(+)/LND(n = 56)	P	Image(-) /LNS(n = 32)	Image(-) /LND(n = 40)	P
Recurrence	34 (54.8%)	56 (58.3%)	0.665	19 (60.0%)	37 (66.1%)	0.576	16 (50.0%)	19 (47.5%)	0.833
Recurrence site in recurrent cases									
LN	21 (61.8%)	19 (33.9%)	0.01	11 (61.1%)	12 (32.4%)	0.043	10 (62.5%)	7 (36.8%)	0.13
Isolated LN	6 (17.6%)	6 (10.7%)	0.358	3 (15.8%)	4 (10.8%)	0.671	3 (18.8%)	2 (10.5%)	0.642
Peritoneal seeding	10 (29.4%)	23 (41.1%)	0.266	5 (26.3%)	16 (43.2%)	0.215	5 (31.2%)	7 (36.8%)	0.728
Liver	11 (32.4%)	19 (33.9%)	1	7 (38.9%)	13 (35.1%)	0.786	4 (25.0%)	6 (31.6%)	0.668
Lung	3 (8.6%)	3 (5.4%)	0.672	3 (15.8%)	2 (5.4%)	0.197	0	1 (5.3%)	0.352

LNS, lymph node sampling; LND, lymph node dissection; Image(+), positive lymphadenopathy on preoperative CT scan; Image(-), negative lymphadenopathy on preoperative CT scan; LN, lymph node

Univariate and multivariate Cox regression analyses of PFS and OS in all patients are shown in Table 4. The multivariate analysis of PFS identified a preoperative CA 125 level >500 U/ml, histologically proven nodal metastasis, and systematic LND as significant factors (P = 0.03, 0.003, and 0.045, respectively). The hazard ratio (HR) of LND for PFS was 0.629 (95% confidence interval [CI], 0.400–0.989). The multivariate analysis for OS identified age and systemic lymphadenectomy as significant factors (P = 0.004, and P < 0.001, respectively), and the HR of LND for OS was 0.250 (95% CI, 0.137–0.456).

**Table 4:** Univariate and multivariate analyses of various factors for PFS and OS

	No. of patients	PFS				OS			
		Univariate analysis		Multivariate analysis		Univariate analysis		Multivariate analysis	
		HR (95% CI)	P	HR (95% CI)	P	HR (95% CI)	P	HR (95% CI)	P
Age, years (continuous)	158	1.016 (0.994-1.039)	0.154			1.043 (1.013-1.075)	0.005	1.044 (1.014-1.075)	0.004
Histology									
Non-serous	28	1 (Reference)				1 (Reference)			
Serous	124	1.226 (0.611-2.462)	0.567			1.072 (0.454-2.530)	0.874		
Tumor grade									
Grade 1	7	1 (Reference)				1 (Reference)			
Grade 2-3	151	1.364 (0.576-3.186)	0.227			1.140 (0.345-3.765)	0.83		
Preoperative CA 125, U/ml									
≤500	58	1 (Reference)		1 (Reference)		1 (Reference)			
>500	94	1.722 (1.030-2.881)	0.038	1.713 (1.053-2.789)	0.033	1.540 (0.782-3.033)	0.212		
Suspected LN metastasis on preoperative imaging studies									
No	69	1 (Reference)				1 (Reference)			
Yes	83	1.326 (0.809-2.171)	0.263			1.461 (0.746-2.860)	0.269		
LN metastasis									
No	55	1 (Reference)		1 (Reference)		1 (Reference)			
Yes	103	2.060 (1.162-3.651)	0.013	2.238 (1.304-3.841)	0.004	1.331 (0.628-2.820)	0.455		
Residual disease									
NGR	39	1 (Reference)				1 (Reference)			
R < 1 cm	113	1.102 (0.604-2.011)	0.752			1.251 (0.498-3.142)	0.634		
Lymphadenectomy									
LNS	58	1 (Reference)		1 (Reference)		1 (Reference)		1 (Reference)	
LND	94	0.613 (0.384-0.979)	0.041	0.629 (0.400-0.989)	0.048	0.310 (0.163-0.588)	<0.001	0.250 (0.137-0.456)	<0.001

PFS, progression-free survival; OS, overall survival; HR, hazard ratio; CI, confidence interval; CA 125, cancer antigen 125; NGR, no gross residual disease; R, residual disease; LNS, lymph node sampling; LND, lymph node dissection

## DISCUSSION

In the current study, we observed a significant improvement in OS and marginally significant improvement in PFS in patients who underwent pelvic and para-aortic systematic LND during primary optimal debulking surgery for advanced-stage ovarian cancer. Moreover, in a subgroup analysis according to the gross lymphadenopathy status as assessed by preoperative CT scanning, significant beneficial effects of systematic LND on both PFS and OS were observed in patients with negative lymphadenopathy on a preoperative CT scan. The favorable efficacy of systematic LND might be attributed to the contribution of this procedure to the detection and removal of occult and chemoresistant lymph node metastases, as inferred from our data regarding the characteristics of subsequent nodal recurrence, the incidence of which might be decreased by LND.

Multidisciplinary treatment, which includes cytoreductive surgery and platinum-based chemotherapy, is the mainstay of management for women with advanced-stage ovarian cancer [16]. Although medical treatment is nearly homogenous, surgical treatment is individualized according to the disease extent and patient characteristics and therefore remains heterogeneous. Moreover, the role of complete lymphadenectomy in a primary staging operation with the intent to gain information regarding prognostic relevance remains under debate by many authors [5, 6, 10, 11, 13, 17–22], and therefore a consensus regarding the therapeutic role of this procedure has not been established, particularly after radical surgery was accepted as standard EOC management.

Previous studies have demonstrated the potential importance of systematic LND for the detection of occult lymph node metastases [5, 11, 18]. In two previous RCTs, patients with EOC who were treated with systematic LND had a higher rate of histologically proven lymph node metastasis when compared with those who underwent macroscopic lymph node removal (22% vs. 9% for early-stage disease; 70% vs. 42% for advanced-stage disease) [5, 11], suggesting a potential increase in the opportunity to detect occult lymph node metastasis via systematic LND in patients with advanced ovarian cancer. This hypothesis was verified by a previous study wherein the reported detection rates of lymph node metastasis in patients with peritoneally advanced ovarian cancer ranged from 48% to 75% [18]. Of note, the data from our study also support this hypothesis (72.9% vs. 53.2%, P = 0.011).

In the present study, the sensitivity and specificity of a preoperative CT scan for the detection of lymph node metastasis were 65.0% and 65.5%, respectively. Among 86 patients with suspected lymphadenopathy on preoperative CT scans, nodal metastasis was histologically proven in 67 patients (77.9%). In addition, the false negative rate, or detection rate of occult lymph node metastasis among patients with preoperative CT scans indicating negative lymphadenopathy, was 50% (36 of 72 patients). This observation suggests that a negative preoperative imaging result on lymph node metastasis could not justify the omission of systematic LND during debulking surgery for advanced ovarian cancer.

Incompletely resected occult lymph node metastases may give rise to chemoresistance, a possibility that is supported by the “pharmacologic sanctuary hypothesis” of poor prognosis in patients with EOC with lymph node involvement. Specifically, this hypothesis suggests that the diminished blood supply of lymph node metastases might promote resistance to chemotherapy [23], and further implies that systematic LND might be a favorable prognostic factor in patients with advanced ovarian cancer who have an increased risk of occult lymph node metastasis [24]. Because ovarian cancer is known to spread simultaneously both intraperitoneally and retroperitoneally, the lymphatic spread of a tumor might persist despite achieving optimal cytoreduction of intraperitoneal dissemination, thus contributing to a poor prognosis [25].

Although our data suggest benefits of systematic LND for patients with optimally cytoreduced advanced ovarian cancer, these data should be interpreted with caution. We attempted to minimize potential bias by accounting for all known prognostic variables associated with both the tumors and patients; however, selection bias regarding patient recruitment cannot be ruled out because of the retrospective nature of this study. Moreover, decisions regarding whether to perform systematic lymphadenectomy were made according to each surgeon's discretion, rather than random allocation or well-defined criteria.

Notably, the prognostic relevance of lymphadenectomy is currently under investigation in the prospective phase III trial “Randomized, Multicentre Trial for Lymphadenectomy in Ovarian Neoplasms” (https://clinicaltrials.gov/ct2/show/NCT00712218). This ongoing study compares the prognostic outcomes associated with systematic lymphadenectomy vs. no lymphadenectomy in patients without macroscopic residual intra-abdominal tumors to assess the efficacy of systematic pelvic and para-aortic lymphadenectomy. This trial is expected to clarify the status of this important issue.

In conclusion, the present study has demonstrated the potential therapeutic value of systematic LND with respect to improved prognosis following the optimal removal of intra-abdominal peritoneal metastases, regardless of the preoperatively suspected lymphadenopathy status on CT scans. This finding might explain the correlation of a lower nodal recurrence rate with systematic LND vs. LNS.

## MATERIALS AND METHODS

### Study design

**Figure 3:**
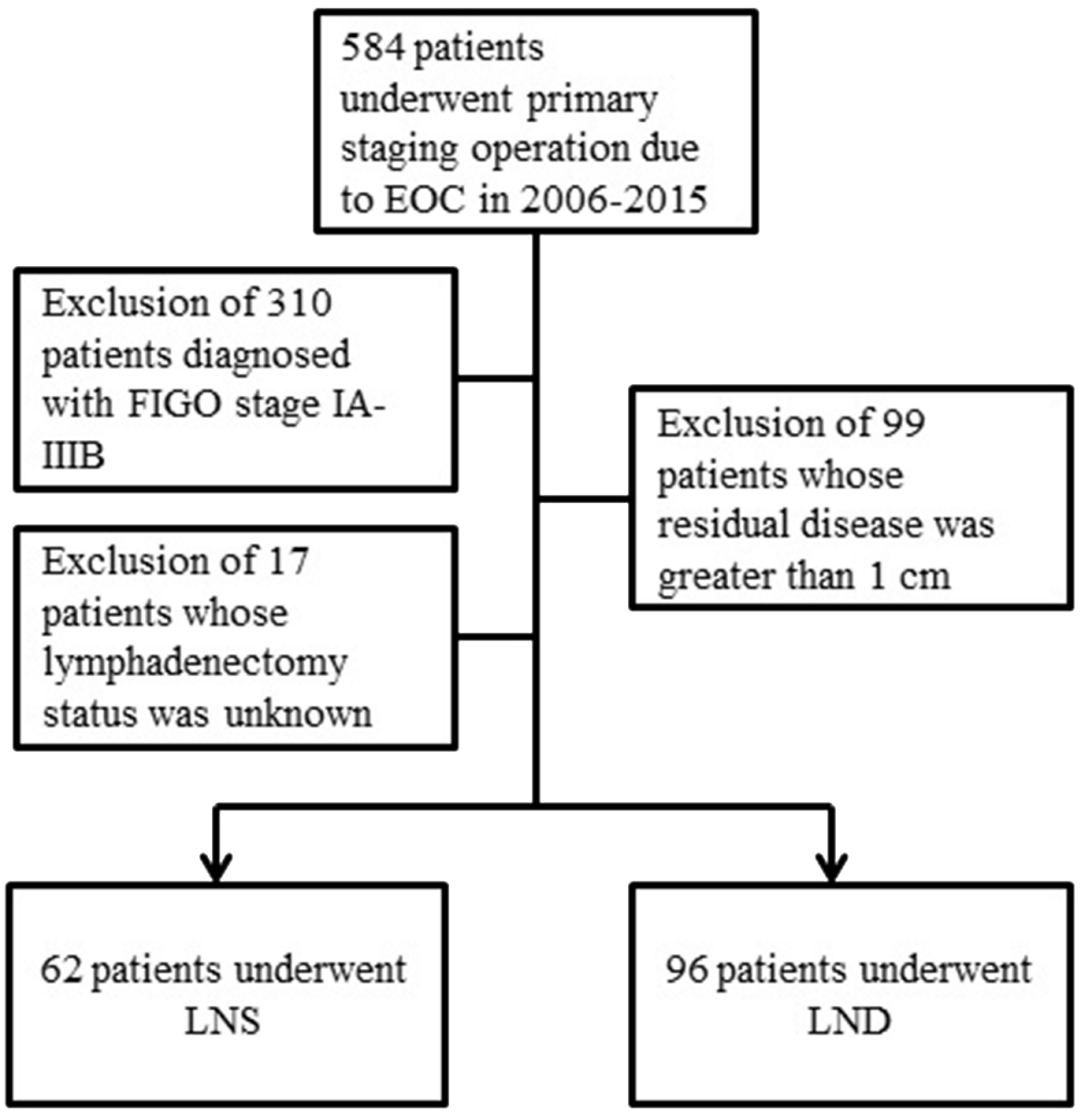
Patient selection diagram LNS, lymph node sampling; LND, lymph node dissection.

A flow diagram of the patient selection process is presented in Figure 3. Patients who were diagnosed with advanced-stage (FIGO IIIC and IV) EOC from January 2006 to December 2015 and underwent optimal cytoreduction, or had residual disease of <1 cm were included in this study. The retrospective study protocol of this study was approved by our Institutional Review Board.

A retrospective chart review was performed to identify all patients who underwent primary debulking surgery, including hysterectomy, bilateral oophorectomy, omentectomy, and retroperitoneal lymph node excision with or without various radical surgeries (e.g., bowel resection, diaphragm resection, peritonectomy) and received adjuvant standard platinum-based chemotherapy. A gynecologic oncology team comprising 5 surgeons at a single institute conducted all procedures, and 2 dedicated radiologists at the same institute reviewed preoperative computed tomography (CT) scan data.

To determine the therapeutic value of systematic LND, patients were classified into LNS and LND groups [11, 26, 27]. Decisions regarding whether to perform LND or LNS were made according to each surgeon's discretion. LNS comprised node sampling and bulky node (≥1cm) removal (total resected node count: <20). LND was defined as systematic lymphadenectomy (total resected node count: ≥20), including both systemic pelvic and para-aortic lymph node dissection. Systemic pelvic LND included the resection of all lymph nodes and fatty tissue between the external and internal iliac arteries from the bifurcation of the common iliac artery up to the circumflex vein and above the obturator nerve. Systemic para-aortic LND included the resection of all lymph nodes and fatty tissue overlying the common iliac artery, vena cava, and aorta anteriorly up to the renal vessels and laterally to the edge of the psoas major muscle. The surgical procedures and characteristics of recurrence and prognosis were compared between the groups.

Additionally, a subgroup analysis was conducted to investigate the role of LND with respect to apparent nodal involvement on a preoperative CT scan. Both groups described above were stratified according to the suspected lymphadenopathy status on preoperative CT scans. Image(+) indicated positive preoperatively suspected lymphadenopathy, defined as the detection of lymph node enlargement (≥1 cm) in the long or short axis on a CT scan. Image(-)was defined as a lack of suspected lymphadenopathy on a preoperative CT scan.

### Statistical analysis

IBM SPSS version 20 for Windows (SPSS Inc., Chicago, IL, USA) was used for the statistical analysis. The Kolmogorov–Smirnov test was used to verify standard normal distributional assumptions. Pearson's chi square test, Fisher's exact test, and the Mann–Whitney U test were used in the univariate analysis. Survival outcomes were determined through a Kaplan–Meier survival analysis. Univariate and multivariate analyses of the effects of various prognostic factors on survival were performed using the Cox proportional hazards model. Multivariate analysis was performed with variables that considered significant in univariate analysis. A P-value of <0.05 was considered statistically significant.
